# Reviewing Physiological Measures of Implicit Consumer Response to Food

**DOI:** 10.3390/foods15193551

**Published:** 2026-10-06

**Authors:** Andres Viadel, Laura Laguna, Amparo Tarrega

**Affiliations:** Institute of Agrochemistry and Food Technology (IATA-CSIC), Agustín Escardino 7, 46980 Paterna, Valencia, Spain; andres.viadel@iata.csic.es (A.V.); laura.laguna@iata.csic.es (L.L.)

**Keywords:** autonomic nervous system, electrodermal activity, EEG, fMRI, consumer, sensory

## Abstract

This review provides information about the contribution of the different physiological and neural methods of measuring consumer responses to food and discusses their potential for improving the understanding of processes underlying food pleasure and choice. A substantial body of work (132 studies published between 2011 and 2026) has recorded consumers’ implicit responses to foods, using physiological (ANS) methods such as Galvanic Skin Response (GSR), Heart Rate (HR), Pupil Size (PS), and facial expressions; and neuronal methods such as Electroencephalography (EEG) and functional Magnetic Resonance Imaging (fMRI). Autonomic Nervous System (ANS) indicators (GSR, HR, and PS) are activated during smelling or eating, with variations across products, likely reflecting the role of the ANS in the anticipatory and alert mechanisms of the body in response to food. However, the analysis of its relationship with explicit liking showed discrepancies depending on the study (product, protocol, context), indicating that they provide information about consumers’ reactions that are different from those explicitly reported. Neuronal measures show differences in brain activity in response to foods, with fMRI identifying regions such as the Orbitofrontal Cortex and Somatosensory Cortex in response to foods. Integration of self-report assessment, implicit behaviour, physiological, and neural responses may provide a deeper understanding of the neural pathways linking sensory perception, autonomic activation, consumer decisions, and food-related pleasure.

## 1. Introduction

Consumer studies in food and nutrition research have traditionally relied on self-reported measures, commonly referred to as explicit methods [1]. Through questionnaires, rating scales, hedonic tests, or emotional profiling techniques, consumers are asked to consciously evaluate products, express attitudes, and report their perceptions. These approaches assume that individuals are aware of the factors influencing their judgments and easily express them. However, decisions can come from the outcome of two different cognitive pathways: a conscious, analytical route that generates explicit responses; and a rapid, automatic route that operates largely outside conscious awareness and generates implicit responses [2]. In some cases, these automatic processes, referred to as implicit responses, could also be determinants of food choice [3], even if the consumer is not aware of them.

In the last two decades, different methodological approaches have been proposed to assess indicators of the automatic response of consumers. Implicit methods are techniques that can measure non-self-reported consumer responses and may serve as indicators of the consumer’s automatic response. On one hand, approaches from a psychological perspective, such as the Implicit Association Test, are able to infer attitudes, preferences, or emotional associations not declared by consumers by measuring reaction time [4,5]. On the other hand, physiological measurements have also been proposed, assuming that they are indicators of the body’s spontaneous and automatic reactions [6]. Physiological indicators commonly used in food studies include Facial Expressions and Autonomic Nervous System (ANS) measures, such as Pupil Size (PS), Heart Rate (HR), and Galvanic Skin Response (GSR). Finally, brain activation measures, such as Electroencephalography (EEG) and functional Magnetic Resonance Imaging (fMRI), have also been used to investigate the neural mechanisms underlying liking, wanting, reward processing, expectations, and food choice.

**Figure 1:**
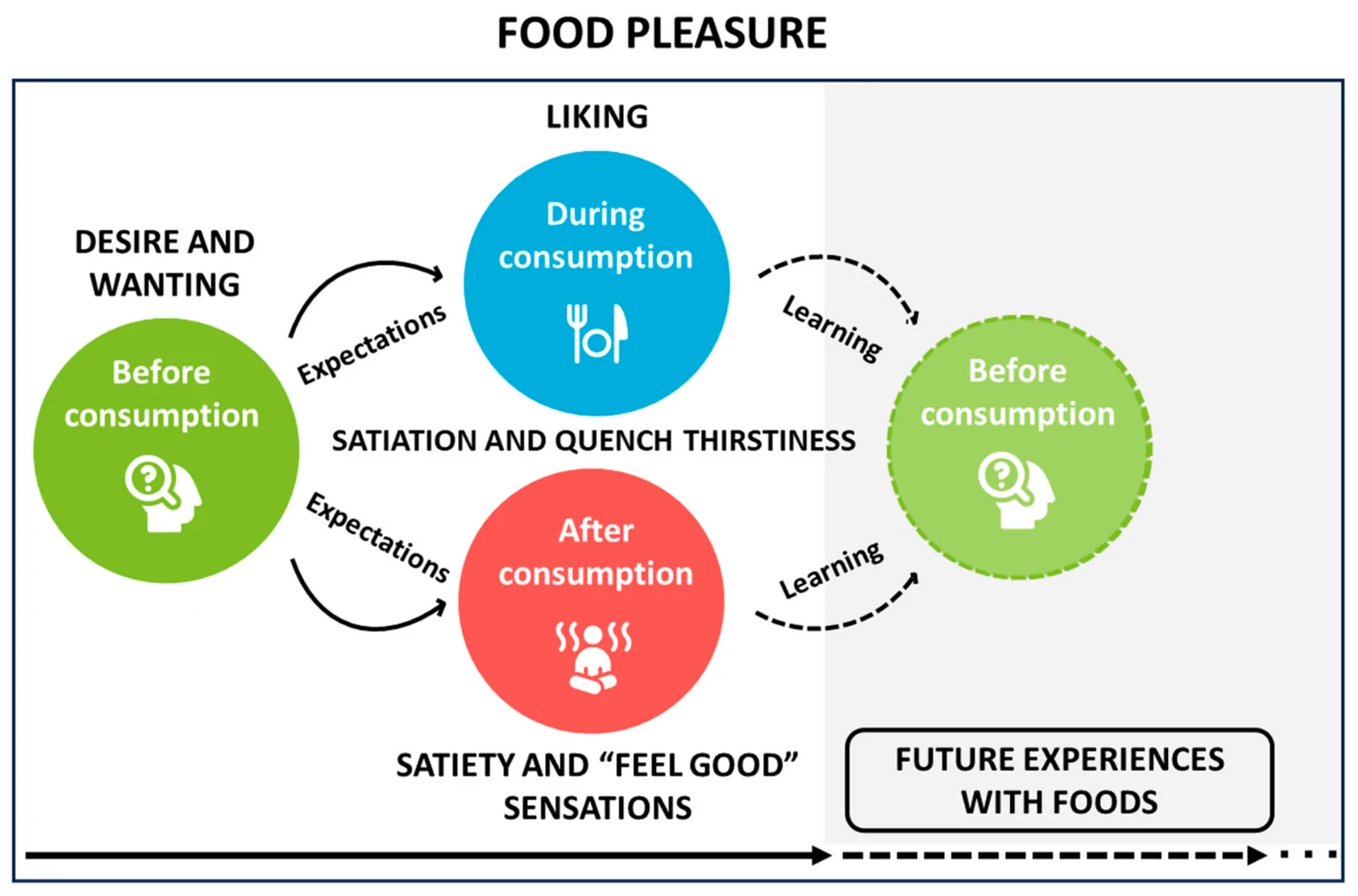
Dimensions involved in food perception and pleasure experience during food consumption.

These implicit processes can be particularly relevant when considering pleasure, one of the major drivers of food choice and consumption. Food pleasure is a multidimensional construct that extends far beyond the sensory enjoyment experienced during eating [7]. As shown in Figure 1, pleasure encompasses anticipatory processes occurring before consumption, hedonic experiences during consumption, and post-ingestive consequences after eating [8,9].

Before consumption, desire and wanting sensations driven by pleasure expectation influence food-seeking behaviour [10]. During consumption, pleasure is mainly expressed as liking, the hedonic appreciation of sensory properties such as taste, aroma, appearance, and texture [11,12]. However, liking is not determined exclusively by sensory input, as it is also shaped by contextual information, expectations, social influences, cultural norms, and individual characteristics [13,14]. After consumption, post-ingestive sensations, including satiation, satiety, thirst quenching, comfort, and other feelings of well-being, contribute to the overall pleasure experience and influence future food preferences through learning mechanisms [15,16].

All these dimensions of food pleasure can be influenced by automatic processes. The sensation of food desire is considered a conscious motivational state, but wanting is considered to run through unconscious processes that can be triggered by external food cues, physiological needs such as hunger, or learned associations acquired through previous experiences [10,17]. Emotional memories associated with familiar foods can evoke feelings of comfort without conscious association to the original experiences [18]. Repeated exposure to foods, brands, or consumption contexts can generate automatic preferences through familiarity and mere exposure effects. Furthermore, food stimuli can activate learned associations, cultural meanings, habits, and reward expectations that bias judgments before conscious evaluation occurs [19]. Physiological states such as thirst, fatigue, stress, or hunger can direct attention toward specific foods without consumers being aware of their influence. Automatic thinking can also facilitate rapid decision-making through mental shortcuts based on previous experiences, allowing consumers to make choices without extensive cognitive effort [20]. Consequently, food preferences and choices can emerge from the interaction between conscious evaluations and unconscious affective, cognitive, and physiological processes. There has been an interest in methods capable of capturing the implicit consumer response to access dimensions of consumer experience that can remain hidden when relying exclusively on self-report measures [21,22], which may perhaps explain discrepancies between what consumers report and what they actually choose, consume, or experience.

In recent years, a growing number of studies have included implicit methods for the evaluation of food in different contexts. Many of them registered explicit responses looking for predictors of emotional reactions or liking. However, implicit methods could also be indicators of homeostatic regulation, anticipatory and alert mechanisms, or other cognitive processes [6]. The analysis of comparative studies including both approaches (implicit and explicit) may help to understand whether similar or different information is obtained from them and to identify the usefulness of implicit information in explaining consumer behaviour. The aim of this study was to examine the contribution of the different physiological and neural methods to measure consumer responses to food and discuss their potential for improving the understanding of processes underlying food pleasure and choice. For that, this review has included 132 research studies published in the last 15 years (between 2011 and 2026) that studied the physiological (GRS, HR, PS, and Facial Expressions) and neuronal responses (EEG and fMRI) to food. As a starting point, the main areas and pathways of the nervous system involved in responses to food are summarised to provide an understanding of how these responses emerge and the potential insights they may offer.

## 2. Nervous System Pathways Involved in Response to Food

**Figure 2:**
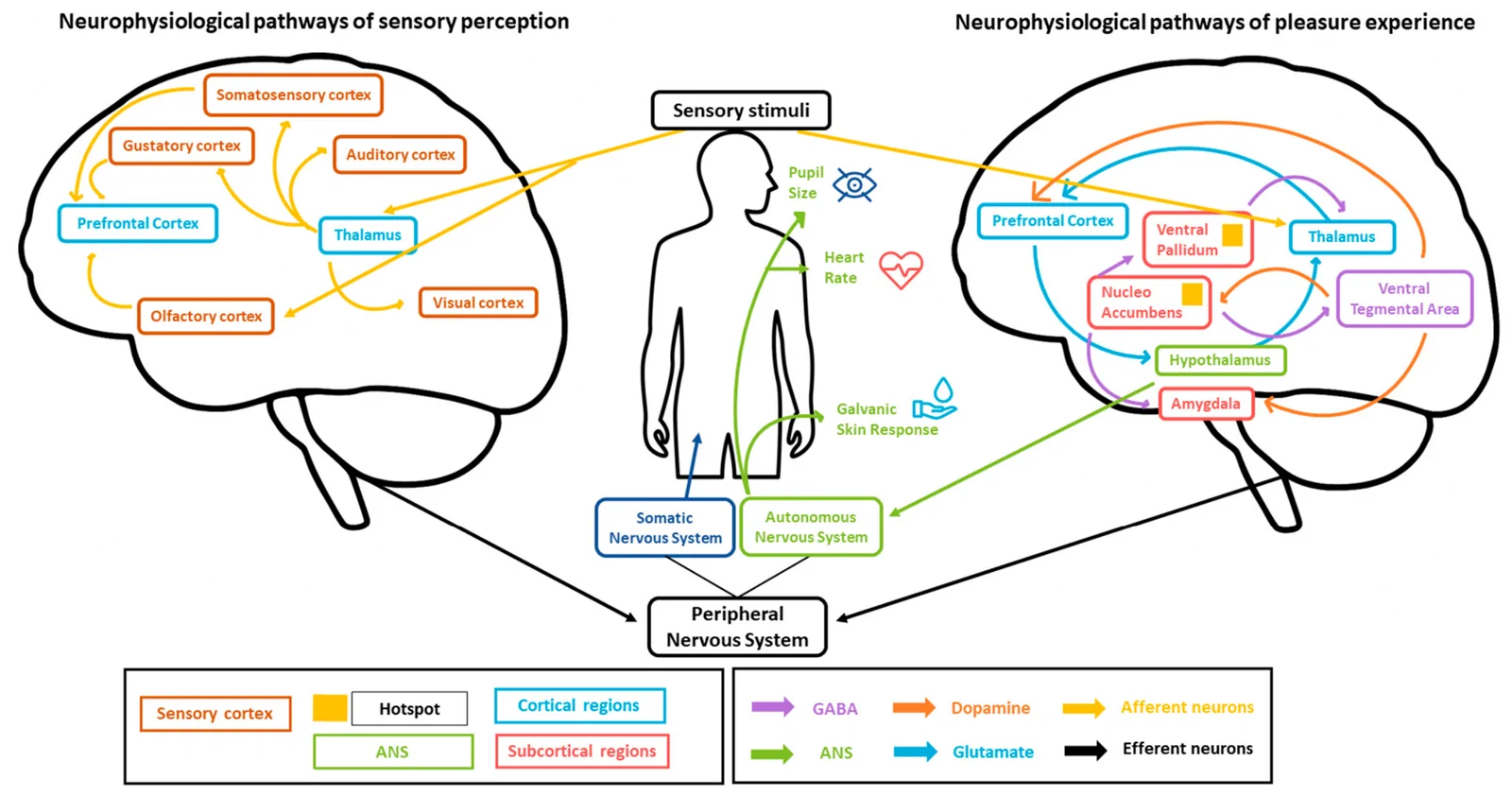
Neurophysiological pathways in the brain and body from sensory stimulation to pleasure during food consumption. The left side shows the neurophysiological pathways of sensory perception of food, and the right side shows the neurophysiological pathways of food-pleasure experience. The boxes represent the different areas of the brain, and the arrows represent the connections involved in the neurophysiological pathways.

Figure 2 illustrates the main neurophysiological pathways and the multiple brain regions involved in the nervous system in response to food. On the left side of Figure 2 are summarised the pathways of the nervous system involved in sensory perception and response to food. Sensory organs are initially activated by food stimuli, which have specialised receptors, such as neuroepithelial cells, that detect external or internal stimuli in different parts of the human body (eyes, nose, ears, and oral cavity) [23]. These sensory receptors convert physical or chemical stimuli into electrical signals. These sensory signals travel through afferent neurons (yellow arrows) to the thalamus, which acts as a relay and distribution centre that transmits information to the different parts of the specific sensory cortex areas (boxes in orange), including the gustatory cortex, somatosensory cortex, auditory cortex and visual cortex. Here, integration of different signals finally enables sensory perception, for instance, building and identifying sensations, such as recognising the sweet taste of candy or the crunchiness of a biscuit. In the case of smell, sensory receptor signals pass directly to the olfactory bulb and from there to the olfactory cortex, bypassing the thalamus.

Simultaneously, some food stimuli activate the hedonic circuit in the cortical and subcortical regions, where dopamine and other neurotransmitters play a fundamental role in generating pleasure. Figure 2 shows, on the right side, the parts and mechanisms involved in the pleasure experience during food consumption. Dopamine (orange arrows) plays a key role in this circuit [24]. Dopamine is produced in the Ventral Tegmental Area (VTA) and released mainly to the nucleus accumbens (NAc), which is a part of the ventral striatum with an important hotspot (NAc Hotspot) [25]. The nucleus accumbens sends gamma-aminobutyric acid (GABA) projections (purple arrows) to the VTA, establishing the circuit NAc-VTA [26]. After activation of the NAc-VTA circuit, the nucleus accumbens sends GABA projections to the Ventral Pallidum, where another major hotspot (VT hotspot) of the brain’s hedonic system is located [10,27]. In addition, the ventral pallidum establishes a connection with the thalamus through GABA projections. VTA is responsible for sending dopamine projections to the amygdala and regions of the prefrontal cortex, which include the Orbitofrontal Cortex (OFC) [26]. These regions are interconnected with the NAc through glutamate projections (blue arrows) that can amplify the sensations of pleasure [10,28].

Both pathways (sensory perception and pleasure) are connected to structures such as the hypothalamus [27], hippocampus and amygdala [29], triggering responses in the Peripheral Nervous System [30]. The main function of the Peripheral Nervous System is to connect the response of the central nervous system to the limbs and organs of the body through efferent neurons (black arrow). The Peripheral Nervous System is divided into the Somatic Nervous System, responsible for voluntary control of skeletal muscles, and the Autonomic Nervous System (ANS), which regulates involuntary physiological processes essential for maintaining homeostasis of the body [31]. ANS response is mainly composed of two branches. Sympathetic Nervous System prepares the body for “fight or flight” responses by increasing alertness and mobilising energy. The Parasympathetic Nervous System promotes “rest and digest” functions by conserving energy and facilitating recovery and digestion. For instance, autonomic responses include changes in heart rate, sweating, pupil size, and digestive activity. A notable example is the cephalic phase response, in which sensory cues associated with food trigger parasympathetic activation and prepare the gastrointestinal system for digestion [32,33].

Considering these main areas and pathways of the nervous system involved in the response to food, the following sections review the physiological and neural measures used to assess implicit consumer responses. For each method, information is discussed regarding its reaction and sensitivity to food stimuli, its relationship with explicit consumer responses, and its strengths and limitations for understanding the neurophysiological processes underlying food pleasure and consumer choice.

## 3. Physiological Measurements of Consumer Response to Food

### 3.1. Galvanic Skin Response

Galvanic Skin Response (GSR), also known as electrodermal activity, is a measure of the electrical activity of the skin [34]. The sympathetic nervous system increases the activity of sudoriparous glands and sweat production, which increases the electrical conductivity of the skin [16]. GSR, as a non-invasive marker, can be measured by just applying an electrical potential between two points of skin contact (fingertips, hands, or feet) and measuring the resulting current flow between them [35]. GSR signal comprises two main components [16]: the tonic level, the slow change of baseline on a time scale and the phasic response, which represents the faster and event-related part of GSR. Among them, researchers focus on the changes in the phasic response that are measured through the Skin Conductance Response, expressed in microSiemens (μS) and inversely proportional to Skin Resistance Response.

Previous studies have studied the GSR of consumers in response to different foods, odours, beverages, and food images (Table 1).

During the observation of food images, most of the studies showed a low GSR response and no significant variation among food product images [51,52,53,54], probably because just observing images of food cannot be a sufficiently strong stimulus to activate a GSR response. Just in some cases, GSR showed to be more activated when observing other visual formats such as advertisements [58], packages [43,44], different products [39] or videos [59], because they probably contained more stimulating visual elements (with movement and appealing designs).

During smelling and tasting, literature has reported that GSR can significantly vary among products or not, depending on the study and the type of product evaluated. For instance, GSR varied significantly among juices [37], beers [36], food odours [38,40], caffeine or sucrose solutions [42], chewing gums [41], and different food categories [39]. However, other studies have not detected significant differences among juices [47], drinks [48], chocolate puddings [49], different food categories [46], and breakfast drinks [38]. Therefore, some studies have reported significant differences in GSR responses among products, whereas others have not.

In the studies that found significant differences among products, the relationship between GSR response to foods and liking has been approached in several studies with different outputs depending on the study. A negative correlation between GSR and self-reported liking has been found when analysing consumer responses to types of juices [37], beers with different sensory properties and alcohol content [36], and pleasant and unpleasant odours [38,40]. Other authors have shown that the GSR is positively correlated with the acceptance of different sweet gums [41] or caffeine or sucrose solutions [42]. Therefore, there is no clear relationship between liking and GSR response. In a recent study from our research group [45], which included 10 food products covering a wide range of liking variation, no significant relationship was found between GSR response and explicit liking.

Changes in GSR intensity could be related to other differences than liking itself, such as familiarity or the intensity of sensory sensations. In fact, it seems that GSR increases for individuals with high neophobia or low familiarity with the product. Specifically, two studies have shown that GSR was significantly higher for neophobic participants when consumers observed food images [53] or tasted different types of hot dogs [56]. De Wijk et al. [39] found that children showed increased GSR responses to disliked foods, whereas adolescents showed decreased GSR responses. Authors suggested that these differences were related to familiarity with the foods; children had limited previous exposure and were therefore less familiar with the products, while adolescents had greater exposure and familiarity, leading to different physiological response patterns. Therefore, most research suggests that GSR is higher for unknown or unbranded products due to novelty. However, Smith et al. [57] showed higher GSR levels when participants evaluated branded rather than unbranded products. This difference likely occurs because the products used in their study were already known to be pleasant and preferred by consumers, whereas GSR typically reacts more strongly to unpleasant or unfamiliar stimuli.

### 3.2. Heart Rate

Heart Rate (HR) is a physiological indicator of heart activity whose changes are mediated by the ANS. The heart is a key organ in the human body, acting as a pump that transfers oxygenated and deoxygenated blood throughout the body. HR is a measure that indicates the number of beats that the heart emits in a period of time and is expressed in units of beats per minute (bpm). Multiple methods and devices can be used for HR registration. In sensory and consumer studies, photoplethysmography (PPG) is one of the most commonly used non-invasive techniques for monitoring heart activity. PPG sensors are attached to the skin (usually on the finger or ear) and indirectly measure changes in blood volume using light [34]. The reflected or transmitted light from the area covered by the PPG light source provides a pulsating signal, but it does not directly record the heartbeat as an electrocardiogram would, which records the electrical heart activity [33]. Furthermore, there are other parameters besides HR, such as peripheral blood volume or HR variability, that could be used to register the activity of the heart [35]. Peripheral Blood Volume is used to record changes in blood flow by recording peripheral vasodilation or vasoconstriction, and Heart Rate Variability is used to record the variation in time between one heartbeat and another [35].

Previous works have studied the HR of consumers in response to different foods, odours, beverages, and food images (Table 2).

During the observation of food products or an image of them, the HR is low and shows small differences among products. For example, HR does not significantly vary among images with different appeal [52,69], or during the visualisation of videos of breakfast meals [67]. During smelling and tasting, the literature has reported that HR can significantly vary among products or not, depending on the study and the type of product evaluated. On the one hand, research has reported differences in HR among taste solutions [42,60], chocolate puddings [49], yoghurts [63], chewing gums [61], different food categories [46], stir-fried products [62], drinks with different carbonation [48], breakfast drinks [50], and odours [38]. On the other hand, HR of participants did not significantly vary among vegetable and fruit juices [37,47], commercial beers with different fermentation [64], chocolates with the five types of tastes [65], and different food categories [66].

In studies showing differences in HR when consumers were eating or smelling different products, the relationship with the sensory properties (texture and flavour) of the product has been analysed. Interestingly, HR changes have been found to be mainly associated with texture. Studies have reported a correlation between HR and the hardness and firmness of the evaluated products. In chewing gums, Farella et al. [61] found that HR during consumption varied significantly for different textures (soft, moderate, and hard). HR was higher during the consumption of the hardest chewing gum, in parallel with muscle activity. Gupta et al. [63] showed that HR was positively related to firmness during the evaluation of yoghurts, with HR higher during the evaluation of yoghurts with crunchies. Other research work studied the effect of masticatory and salivary capabilities of elderly participants on HR response during food consumption, showing that HR was lower for participants with good oral capabilities than for those with low salivary or masticatory capabilities, who required extra effort during the eating task [46]. De Wijk et al. [62] compared the HR response of consumers during consumption at the sensory room and at home, showing that HR was higher during consumption at home than in the sensory room. These authors suggested that the higher HR observed during eating at home compared with the sensory room indicates that foods were consumed with greater effort and chewed more vigorously in the home environment.

The relationship between HR response to foods and liking has been approached in several studies with different outputs depending on the study. Most studies did not find a significant relationship [45,49,50,62,66] or a weak correlation [41]. In a few studies, a negative correlation between HR and liking was found for different solutions with sucrose and caffeine [42], solutions with the four basic tastes [60], and fish and orange odours [38]. Liking was positively correlated with HR in a study on yoghurt; however, in this case, liking variation coincided with differences in the hardness of yoghurt, which could be the origin of HR changes [63]. Thus, there is no clear relationship between explicit liking and familiarity with HR response. Furthermore, there is no consistent evidence of a direct link between HR and familiarity or neophobia. Raudenbush and Capiola [53] showed that HR changes during food picture observation were related to participants’ food neophobia. Individuals with higher neophobia showed a higher HR than those with low neophobia. However, Torrico et al. [66] reported no significant correlation between HR and familiarity.

### 3.3. Pupil Size

Pupillometry is a physiological technique used to measure changes in Pupil Size (PS), which is regulated by the ANS. Variations in PS, manifested as dilation or constriction, reflect the dynamic balance between sympathetic and parasympathetic activity [70]. In humans, PS typically ranges from approximately 2 to 8 mm [71]. These changes are mediated by the coordinated action of two iris muscles: the dilator muscle, associated with pupil enlargement, and the sphincter muscle, responsible for pupil constriction [72]. In food science, pupil responses are commonly assessed using infrared eye-tracking systems. Previous studies have explored how PS varies in response to different food odours and images (Table 3).

On the one hand, significant differences were observed among odours (mint, orange, mushroom, bread, vanilla, coconut, strawberry, raw potato, oil paint, and wet earth) [76] or (rose, lemon, rotten eggs, and CO_2_) [77], and these two studies found a positive correlation between odour intensity and PS, indicating that olfactory stimulation may increase the activation of the sympathetic system, resulting in greater PS. Second, other studies reported significant variations in PS depending on the type of food image observed. For example, differences in PS have been found among images of web shops [74], cosmetic packages with different colours [80], yoghurt packages with different types of logos [73], images with different appeal [52], videos with different formats (formal and TikTok) and messages [59], and pleasant and unpleasant images [79].The studies that found significant differences among food products (odours and images) have attempted to correlate this response with liking, preference or craving. In an interesting way, the studies found a positive correlation between PS and liking or craving during the evaluation of images of web shops [74], yoghurt packages with different logos [73], and images with different appeals [52], desserts in a real-life buffet [75] and high- and low-calorie food images [84]. However, other studies found that PS was not correlated with liking during the evaluation of 10 food products [45] or that PS was activated in response to pleasant and unpleasant images compared to the neutral ones [79]. Therefore, explicit liking does not appear to be directly related to the PS response. Even so, it should be noted that PS response to images must be interpreted considering that it can be highly affected by image brightness or darkness [79].

Furthermore, PS appears to be influenced by individual consumer characteristics, particularly body weight status, product familiarity, and hunger state. Akcay et al. [83] investigated the effect of body weight on PS responses during the evaluation of auditory food-related words and found that individuals with obesity exhibited significantly smaller PS responses than normal-weight participants. Similarly, Graham et al. [84] explored PS responses to food images varying in caloric content and taste category (sweet and savoury) across participants with different body mass index (BMI). Their results showed that participants with a high BMI exhibited greater PS responses to high-calorie sweet images than to high-calorie savoury images, whereas no such differences were observed in participants with a low BMI.

Beyond body weight status, PS has also been associated with consumers’ previous experience and involvement with food products. For example, Franzen et al. [82] reported greater PS responses to images of familiar products compared with unfamiliar products, suggesting that prior exposure and product knowledge may modulate attentional and emotional processing. Another factor consistently affecting PS is hunger status. Rebollo et al. [52] examined participants under fasted and fed conditions and found that, during the fasted state, highly appealing food images elicited larger PS responses than low-appeal and non-food images. These findings support the idea that the relevance of food stimuli is heightened during hunger. Likewise, Binder et al. [81] investigated children’s responses to candy images while considering household restrictions on candy consumption. The authors found that PS was higher in children who were not allowed to consume candy at home than in those who were allowed to eat candy, further suggesting that food-related motivations and prior dietary experiences can shape pupillary responses to food cues.

## 4. Facial Expressions Measurement of Consumers to Food

Facial expressions can be voluntary (e.g., smiling on purpose) or involuntary (e.g., automatic expressions of pain or happiness) motor responses that originate in the brain and are executed using facial muscles. The most common emotions extracted from facial expression measurements are: happy, sad, angry, surprised, scared, disgusted, and neutral. Facial expressions have been measured and recorded using different techniques from two perspectives: studying video and coding emotions or muscle activation and recording by Electromyography (EMG). Most methods currently used are based on the Facial Action Coding System. This system classifies expressions based on individual muscle movements, called Action Units. For example, raising the corners of the mouth (smile) or raising the inner eyebrows (worry or surprise) [85]. Coding can be done manually by trained or expert analysts, but in recent years, the development of video-based automatic coding systems has radically changed facial expression analysis by reducing the time of analysis and increasing accessibility to facial expression analysis [86]. Tools such as FaceReader^®^ or Affectiva^®^ track key points on the face and compare them with predefined models to detect facial expressions and correlated emotions [86]. EMG measures the electrical activity of the facial muscles using electrodes placed on the skin. The connection between facial actions and muscles is associated with specific emotions. For instance, the orbicularis oculi muscle is related to raised cheeks (happy), and the corrugator supercilia with frowning (angry, sad, or disgusted) [87]. Most studies in food science use video-based automatic coding systems to measure facial expressions of consumers.

Previous literature has studied how facial expressions of consumers change in response to different food products during smelling, observing or eating (Table 4).

During smelling food odours, unpleasant fish odour triggered fewer neutral expressions and more disgusted and angrier expressions than orange odour [38]. In another study, a pleasant orange odour was associated with neutral and surprised expressions and with less disgust than less pleasant odours with higher orange concentrations [40]. He et al. [90] conducted a study in which pleasant odours (chocolate, cucumber, orange, and apple) were associated with fewer expressions of disgust, fear, surprise, anger, sadness, but also happiness. During the observation of images of cold lunch items, small differences were found among the facial expressions of sadness, disgust, and anger, with these being higher for disliked food than for liked foods [39]. In another case, patients with swallowing disorders showed variation in happiness when they looked at hospital food trays with pureed meals, with increased happiness for meals with adapted textures and improved presentation [93]. Torres et al. [92] evaluated the changes in consumers’ facial expressions when they observed images of different lettuce cultivation systems. Results showed that the highest levels of surprise were evoked by images of indoor vertical farming and urban gardens, which were the two least well-known agricultural systems among consumers. However, in other studies, no significant differences in facial expression were found when participants looked at four packages of fish burgers that changed in colour and format [54], nor with different food categories that varied in fat, sugar, or taste [51] or videos of breakfast meals with messages about food hygiene and quality [67].

Although facial expressions have been theoretically designed to capture spontaneous emotional responses during food evaluation, the intensity of facial reactions elicited by food stimuli is often low, resulting in only small differences among products, images, or odours. This may be because the facial expressions triggered by foods are generally less intense than those elicited by other stimuli (e.g., fear or joy). Also, facial responses may be influenced by social masking, whereby participants consciously or unconsciously regulate the expression of their emotions according to social norms or experimental settings.

Other studies have also focused on facial expressions while eating. This poses a challenge in isolating the muscular movements involved in eating from those involved in expressing emotions. The literature has reported that, depending on the type of product, some facial expressions were higher than others, but there is no clear trend or reasoning. For example, when drinking yoghurt and fruit drinks, the authors reported significant differences among happy, sad, angry, surprised, scared, and neutral emotions [50]. Other researchers using fruit and vegetable drinks reported facial expressions of happy, disgusted, neutral, sad, and a sum of negative expressions [37]. Danner et al. [88] reported that angry, disgusted, and neutral emotions significantly varied among orange juices. Another study showed that sad, surprise, and scared varied among different stir-fried products [62]. Therefore, there is no clear consensus regarding the observed differences across studies, and some studies have even reported no significant differences, such as those involving chocolates representing the five basic tastes [65] and two energy drinks [91].

Some studies that incorporated the measurement of facial expressions attempted to correlate these expressions with self-reported emotions. There is no evidence of a direct and strong relationship between facial expressions and self-reported emotions [40,90,91]. In addition, there is no relationship between facial expressions and liking for beers produced with different fermentation [64] or drink yoghurts and fruit drinks [50]. Only a few studies reported weak relationships of liking with negative emotions during the evaluation of different gums and odours [41], juices [88], and different beverages [95].

Studies investigating facial expressions in relation to consumer attitudes have mainly indicated that there is no direct or clear relationship between facial expressions and familiarity. Torrico et al. [66] compared the effect of consumer familiarity on facial expressions towards ten foods, and they showed that only disgust and surprise were weakly correlated with familiarity. Similarly, De Wijk et al. [94] compared the effects of familiarity on facial expressions towards soy sauces, and they showed that facial expressions were weakly affected by familiarity.

## 5. Neuronal Measurements of Consumers’ Response to Food

### 5.1. Electroencephalography

Electroencephalography (EEG) records the electrical brain activity using surface electrodes placed on the scalp. Specifically, EEG registers the electrical activity transmitted from the dendrites to the axon of neighbouring neurons in the brain in the form of waves. The number of electrodes ranges between 3 and 256, but the most common is 10–20 electrodes. The positioning and naming of the electrodes followed the International Naming System. The electrodes are named according to the brain region where they are placed (i.e., F = Frontal, C = Central, P = Parietal, T = Temporal, or O = Occipital), and a number depending on the distance from the midline [96]. Oscillatory signals in brain activity can be described using three defining characteristics: frequency, power, and phase [97]. Frequency is the number of oscillations per unit time. EEG focuses on oscillations between 1 and 80 Hz. Five frequency bands were discerned: delta (0.5–4 Hz), theta (4–8 Hz), alpha (8–13 Hz), beta (13–30 Hz), and gamma (>32 Hz). For example, beta waves represent a normal alert, concentration, and conscious state; alpha waves represent physical and mental relaxation; theta waves represent reduced consciousness; and delta waves represent deep sleep [98]. Second, power refers to the energy amount in specific frequency bands expressed in microvolts (µV). Third, oscillation phase is the point in the oscillation at a specific moment in time, expressed in degrees or radians (rad), and ranges from −π to π rad (−180° to 180°).

Previous literature has studied how neuronal activity of consumers, measured with EEG, changes in response to food (Table 5). Most of the studies showed that brain response is activated during food evaluation tasks, whether under visual, olfactory, or eating conditions. Different frequency bands or regions are activated depending on the stimuli, which are interpreted as body consciousness, meditation, relaxation, and pleasantness. Alpha and beta seem to be the most discriminative bands reflecting the brain’s response to food stimuli. Most studies have indicated that alpha was significantly higher in the frontal and temporal regions during the mastication of chewing gum than in the rest condition [99,100]. However, the effect of mastication on beta remains unclear. Allen et al. [101] showed that beta was significantly higher in temporal and frontal areas during chewing gum mastication compared to the rest period. Aung et al. [102] investigated the effects of cold and hot sprouted wheat drinks on brain activity, showing that after drinking hot beverages, delta and theta waves increased compared to cold drinks, which could be related to relaxation and calmness.

Furthermore, it seems that brain activity is triggered not only by chewing, but also by different tastes and flavours. During the evaluation of tastants, the alpha wave was higher at the occipital, left, and right parietal lobes [105]. In agreement with this, Wu et al. [106] found that alpha activity in the frontal, parietal, and occipital regions could discriminate between different umami compounds, showing that alpha was higher for disodium succinate and disodium inosine monophosphate than for monosodium glutamate in the frontal, parietal, and occipital regions. In another study, Wang et al. [107] compared different types of sweet tastants and showed that alpha significantly increased for erythritol and decreased for sucrose compared to water. Labbe et al. [104] showed that alpha and beta were higher in cortical areas after drinking different water ice with citrus-flavoured and sucrose compared to only water ice. Morinushi et al. [99] indicated that beta was activated during the mastication of the flavoured chewing gum, but was not activated for the unflavoured chewing gum.

Changes in neuronal activity have been observed during odour evaluation. Murao et al. [103] showed that smelling tea, compared to smelling water, significantly increased alpha and beta wave activity in the frontal and occipital regions, respectively. Morinushi et al. [99] indicated that smelling different odours led to an increase in the alpha and beta waves and that delta and theta activity did not significantly vary. Martin [109] measured brain activity to different odours, showing reductions in theta activity for chocolate and spearmint odours compared with the blank and other odours (almond, cumin, strawberry, and vegetable). Authors associated this reduction in theta activity with relaxation in response to the two odours.

Other authors have studied changes in the EEG during food picture visualisation. In most cases, there have been attempts to relate the bands or areas of the brain that are activated to product preference or acceptability. Tashiro et al. [110] reported that when participants viewed food images, beta was significantly higher in response to disliked foods than to favourite foods, and no differences were observed between high- and low-calorie foods. In a study on EEG response towards comfort or daily foods, alpha activation was higher for comfort or indulgent food (chocolate) than daily food (rice), and it was also higher for foreign than local food [108]. Lim et al. [113] showed that brain activity varied in response to four meal images with different dishes and quantities. For liked meals, activity in the anterior insula, fusiform gyrus, praecuneus, ventromedial prefrontal cortex, and medial OFC increased, whereas activity in the dorsolateral prefrontal cortex decreased. Songsamoe et al. [98], who reviewed studies on EEG responses to food, interestingly showed that food acceptability is reflected in hemispheric asymmetries. Increased EEG power in the left hemisphere indicates motivational tendencies towards acceptance, whereas increased activation in the right hemisphere indicates rejection.

Studies investigating EEG responses in relation to consumer attitudes have found differences depending on consumer neophobia. Stuldreher et al. [111] showed that the Late Positive Potential (LPP) amplitude of consumers when observing foods was higher for those with food neophobia, not only for unfamiliar food images but also for the familiar ones. Alpha was higher for foreign (non-familiar) than local food products [108]. The influence of participants’ weight status on their neuronal response to food using EEG has also been studied. Beta was higher in the central regions for high-calorie food in the obese group than in the normal-weight group [112].

### 5.2. Functional Magnetic Resonance Imaging

Functional Magnetic Resonance Imaging (fMRI) is a neuroimaging technique that allows for the study of activity of the different brain regions in real time. The basic principle behind this technique is the blood oxygen level-dependent (BOLD) effect. This effect takes advantage of the difference in the magnetic properties of oxygenated and deoxygenated haemoglobin. When a brain region is activated, neurons require more oxygen. To meet this demand, blood flow to that area increases, changing the ratio of oxygenated to deoxygenated blood, which is detected by fMRI as a BOLD signal [114,115].

Previous literature has studied how neuronal activity of consumers, measured with fMRI, changes in response to food (Table 6). Most studies have shown that brain response is activated during food evaluation tasks, whether under visual, olfactory, or eating conditions.

Different brain regions are activated depending on the stimuli. Luo and Han (2023) [125] have reviewed 52 neuroimaging studies that investigated participants’ neural responses during food evaluation using fMRI. The review reported consistent activation of the orbitofrontal cortex (OFC), amygdala, insula, and cingulate cortices during food evaluation [125]. The OFC is thought to be the brain region most likely to be associated with food pleasantness. For example, studies showed the activation of the OFC towards high-fat foods compared to low-fat foods in milkshakes [116,117,118]. Doornweerd et al. [120] showed higher activity in the OFC during watching food images compared with the non-food ones. Small et al. [119] showed that pleasure-specific responses were observed in the OFC, with the right caudolateral OFC responding preferentially to pleasant compared to unpleasant taste, despite the intensity. Also, other studies on neuronal responses reported activation in the OFC in response to savoury food odours [121] and activation in the amygdala and OFC in response to odours [122]. Furthermore, other studies reported brain regions activated by food-pleasant stimuli. For instance, brain activity in cingulate cortices [116,126] or in the dorsal striatum and mediodorsal thalamus [127] has been associated with food pleasantness. Other studies have shown that neural responses to pleasant food stimuli also depend on the state of the consumer (eucaloric and overfeeding). For instance, Cornier et al. [128] reported that, in a eucaloric state, pleasant food images elicited greater activation of the inferior temporal visual cortex, posterior parietal cortex, premotor cortex, hippocampus, and hypothalamus compared with an overfeeding condition. These findings suggest that metabolic status modulates the neural processing of rewarding food.

Previous literature investigated brain activation in response to aversive or disgusting food stimuli, showing increased activation of the insula and amygdala [129]. Also, Wolfe et al. [124] showed that unfamiliar foods showed significant activation in parietal lobe areas and insula compared to familiar foods. However, these same unfamiliar foods with product name labels had more activation in the prefrontal areas. Royet et al. [123] evaluated how the acceptability of cheese (anti-cheese and pro-cheese) of each consumer affects their neuronal response during their consumption. Neuronal response showed higher activation of the internal and external globus pallidus, VTA, and substantia nigra during consumption in the anti-cheese group than in the pro-cheese group.

## 6. Insights and Future Directions

This review has examined the contribution of the different physiological and neural methods used to measure consumer response to food to discuss their potential for improving the understanding of processes underlying food pleasure and choice. Overall, the reviewed studies show that consumers’ ANS responses are activated in reaction to food and, in some studies, also discriminate among food products. Furthermore, for all the ANS parameters considered (GSR, HR and PS), the analysis of their relationship with explicit liking showed discrepancies (positive, negative, or non-significant correlations) depending on the study (product, protocol, context), indicating that they provide information about consumers’ reactions that is different to the one explicitly reported. However, it is still a challenge to interpret and obtain additional insights from ANS parameters data. Anticipatory or alert responses are automatic responses that could be reflected by the ANS system due to its role in maintaining body homeostasis in response to external stimuli and internal status. On one hand, the cephalic phase response includes ANS activation to food cues, reflecting the body’s direct anticipatory preparation of the digestive and metabolic systems for the incoming nutrients [32,130]. For instance, HR response was shown to be activated by the chewing work that is needed to eat or drink the product during the consumption of harder products [61,63]. These findings suggest that HR responses may not only reflect oral motor effort, but also the activation of anticipatory mechanisms of the ANS, which prepare the body for food consumption and the physiological regulation associated with eating. On the other hand, the ANS system also regulates alert reactions that can be activated by specific sensory stimuli. As it has been observed, GSR and PS showed to respond to high intensity of specific sensory stimuli of olfactory receptors [76,77] or taste receptors (sucrose and bitterness solutions) [42,60]. The alert response is also supported by the fact that GSR is affected by unfamiliarity, GSR increases for individuals with high neophobia or low familiarity with the product [53,56]. Brain regions such as the insula and amygdala have been consistently activated in response to food neophobia, which are regions involved in the pathway of ANS response [124,129].

Future research should therefore focus on disentangling whether these responses primarily reflect physiological preparation, homeostatic regulation, alert mechanisms, or other automatic cognitive processes elicited by food stimuli. In this regard, explicit measures alone are unlikely to clarify the underlying meaning of ANS activation. Integrating ANS recordings with other implicit measures, such as Implicit Association Tests, could help to discern if there are cognitive processes associated with these ANS responses [5]. Likewise, combining ANS measurements with neuroimaging techniques may provide a better understanding of the neural mechanisms underlying autonomic activation and reveal whether different ANS parameters are linked to distinct brain networks [131]. The integration of neuronal techniques (fMRI and EEG) into consumer food research is still in its early stages of development, but these methods are promising for providing a deeper understanding of the neural pathways involved in food perception and acceptance. To date, fMRI has already identified the brain regions involved in sensory perception, including the somatosensory cortex, cerebellum, pons, middle insular cortex, and amygdala [119], as well as areas associated with pleasant sensations, such as the OFC or the limbic system [116,125]. Furthermore, advances in the measurement of brain activity are expected to provide better distinction among the conscious and automatic processes implied in consumer decisions. An interesting neuroimaging study [132] shows the brain areas involve in unconscious and automatic process (midbrain, ventral striatum) and the cortical regions involved with conscious awareness (OFC, parietal cortex).

The discrepancies among studies may be explained by several methodological factors, including the relatively small sample sizes (often lower than 40) often used in experimental studies, the limited number of food samples (typically 2–4), multiple factors acting simultaneously (e.g., temperature, sensory properties, familiarity, context), and discrepancies and differences in study designs and measurement protocols (e.g., period of evaluation, intervals between stimulus). In addition, the implicit techniques reviewed present several methodological limitations. ANS measures such as GSR, PS, and HR are highly sensitive to participant movements and also PS response is influenced by external factors such as image brightness or experimental display conditions. Neurophysiological techniques also have important limitations. EEG studies are prone to artifacts caused by blinking, chewing, and body movements, require complex signal-processing procedures, and provide relatively low spatial resolution, making it difficult to accurately identify specific brain regions involved in food perception. Likewise, neuroimaging techniques are associated with high economic costs, artificial testing environments that differ considerably from normal eating situations, and practical difficulties in evaluating food products, such as solid foods. Future research should employ larger number of participants, a wider range of food products, and more ecologically valid experimental settings to better capture the complexity of food experiences. Such studies would provide deeper insights into the integration of sensory, autonomic, and neural processes underlying food perception, pleasure, and consumer decision-making.

## 7. Conclusions

This review highlights that implicit methods, including physiological and neural measures provide information that can be valuable because they differ from explicit consumer responses, but at the same time they are difficult to be interpreted. Autonomic indicators (GSR, HR, and PS) appear to respond primarily to the type, intensity, and novelty of sensory stimuli, likely reflecting the role of the ANS in anticipatory and alert mechanisms triggered by food, and not showing a clear and consistent relationship with liking. Specifically, GSR activation has been found to be mainly associated with taste intensity and familiarity and HR changes have been found to be mainly associated with texture. The intensity of facial expressions elicited by food stimuli is often low, resulting in only small differences among products, and their potential may be highly biased during food consumption due to mastication or oral activity. Among neural measures, EEG offers a relatively accessible approach to studying cognitive and affective responses to foods, whereas fMRI provides detailed information on the brain regions involved in food perception and reward processing, despite its high cost and limited ecological validity. Given its ability to identify the neurophysiological pathways and brain networks underlying food-related responses, fMRI may represent the most valuable approach for improving our understanding of the neural basis and meaning of autonomic responses. According to this review, implicit methods can provide additional information of consumers response to food that differ to the one reported by them and more related to individual feelings and sensations than differences among food products. Taking into account this perspective can help future research to disentangle the meaning of implicit measures on consumer response to food.

## Figures and Tables

**Table 1:** Effect of food products and consumer traits on the Galvanic Skin Response (GSR) of consumers.

Factors	Observed Effects and Relations with Galvanic Skin Response
Tasting commercial beers (***n*** = 128) [36]; tasting fruit and vegetable juices (***n*** = 81) [37]; smelling food odours (***n*** = 26) [38]; tasting different food categories (***n*** = 31) [39]; food odours (***n*** = 28) [40]	GSR negatively correlated with liking during tasting or smelling
Sweet gums and odours (***n*** = 120) [41]; sucrose and caffeine solutions (***n*** = 32) [42]	GSR positively correlated with liking during tasting or smelling
Looking pharmaceutical packages (***n*** = 40) [43]; looking food packages (***n*** = 83) [44]	GSR negatively correlated with liking during visualisation
Tasting different food products varying in liking (***n*** = 85) [45]	GSR was not correlated with liking
Tasting different food categories (***n*** = 94) [46]; tasting different juices (***n*** = 86) [47]; drinking beverages with different carbonation (***n*** = 15) [48]; tasting chocolate puddings (***n*** = 28) [49]; tasting drink yogurts and fruit drinks (***n*** = 19) [50]; smelling different products (***n*** = 85) [45]	GSR did not significantly vary among products during tasting or smelling
Looking food images with different fat content (***n*** = 100) [51]; food images with different appeal (***n*** = 29) [52]; different food images (***n*** = 23) [53]; fish burger packages (***n*** = 40) [54]; looking images of different food products (***n*** = 85) [45]	GSR did not significantly vary among food images
Chewing or oral activity (***n*** = 94) [46]; tasting meals and wines (***n*** = 30) [55]; looking, smelling and eating products (***n*** = 31) [39]	Higher GSR during smelling, chewing or oral activity
Consumer familiarity (***n*** = 31) [39]	GSR depended on the degree of familiarity
Food neophobia (***n*** = 23) [53]; food neophobia (***n*** = 153) [56]	GSR positively correlated with neophobia
Branded and unbranded food (***n*** = 48) [57]	Higher GSR for favourite food brand than the same food but unpackaged

**Table 2:** Effect of food products and consumer traits on the Heart Rate (HR) of consumers.

Factors	Observed Effects and Relations with Heart Rate
Tasting sucrose and caffeine solutions (***n*** = 32) [42]; tasting solutions (***n*** = 34) [60]; food odours (***n*** = 26) [38]	HR negatively correlated with liking during tasting or smelling
Tasting sweet gums and odours (***n*** = 120) [41]	HR positively correlated with liking during tasting or smelling
Chewing gums with different hardness (***n*** = 10) [61]; tasting stir-fried products (***n*** = 32) [62]; tasting yogurts (***n*** = 62) [63]	HR positively correlated with hardness
Tasting drink yogurts and fruit drinks (***n*** = 19) [50]; food products varying in liking and familiarity (***n*** = 85) [45]	HR was not correlated with liking during tasting or smelling
Tasting commercial beers (***n*** = 30) [64]; tasting chocolates with different tastes (***n*** = 45) [65]; tasting vegetable and fruit juices (***n*** = 86) [47]; tasting vegetable and fruit juices (***n*** = 81) [37]; tasting different food categories (***n*** = 60) [66]	HR did not significantly vary among products
Food images with different appeal (***n*** = 29) [52]; breakfast meals videos (***n*** = 40) [67]	HR did not significantly vary among food images or videos
Smelling food odours (***n*** = 26) [38]	HR positively correlated with intensity
Tasting different food categories (***n*** = 94) [46]; drinking beverages with different carbonation (***n*** = 15) [48]; chewing gums (***n*** = 10) [61]; drinking beverages (***n*** = 17) [68]; tasting and smelling different food categories (***n*** = 85) [45]	Higher HR during smelling, chewing or oral activity
Consumer familiarity (***n*** = 60) [66]; (***n*** = 85) [45]	HR not correlated with familiarity
Food neophobia (***n*** = 23) [53]	HR positively correlated with neophobia
Vegetarian and omnivorous (***n*** = 86) [69]	HR does not vary between vegetarians and omnivores

**Table 3:** Effect of food products and consumer traits on the Pupil Size (PS) of consumers.

Factors	Observed Effects and Relations with Pupil Size
Yogurt packages (***n*** = 89) [73]; food images (***n*** = 63) [74] observation of dessert products (***n*** = 6) [75]; food images with different appeal (***n*** = 29) [52]; evaluation of food images during fed state (***n*** = 29) [52]	PS positively correlated with liking
Food odours (***n*** = 39) [76]; odours (***n*** = 21) [77]	PS positively correlated with odour intensity
Pleasant and unpleasant food images (***n*** = 27) [78]; pleasant and unpleasant food images (***n*** = 25) [79]	Higher PS to pleasant and unpleasant images than neutral images
Cosmetic packages (***n*** = 25) [80]; food products varying in liking and familiarity (***n*** = 85) [45]	PS varied among products but was not correlated with liking
Mandarins and gums (***n*** = 68) [81]	PS did not vary among products
Consumer familiarity (***n*** = 17) [82]	PS was higher with images of familiar food compared to unfamiliar foods
Obesity and non-obesity (***n*** = 63) [83]	Obese had lower PS to auditory food words
Weight status (high and low BMI) (***n*** = 61) [84]	High BMI group showed lower PS to images of high caloric sweets than the savoury
Allowed or not to consume candy at home (***n*** = 68) [81]	PS was higher for those that were not allowed to consume candy at home

**Table 4:** Effect of food products and consumer traits on facial expressions of consumers.

Factors	Observed Effects and Relations with Facial Expressions
Tasting fruit and vegetables juices (***n*** = 81) [37]; orange juices (***n*** = 75) [88]; drink yogurts and fruit drinks (***n*** = 19) [50], semi-liquid meals (***n*** = 24) [89], stir-fried products (***n*** = 32) [38,62]; food odours (***n*** = 26) [38]; food odours (***n*** = 28) [90]	Most facial expressions varied among products but showed low intensity
Orange and fish odour (***n*** = 26) [38]	Some facial expressions correlated with liking but showed low intensity
Tasting chocolates with different tastes (***n*** = 45) [65], energy drinks (***n*** = 47) [91]	Facial expressions did not vary among products and with low intensity
Odour intensity (***n*** = 26) [38]	Intense odours produced stronger facial expressions
Different food categories (***n*** = 31) [39]; images of lettuce cultivation system (***n*** = 105) [92]; images of hospital trays with pureed meals (***n*** = 14) [93]	Some specific facial expressions varied among food images, but with low intensity of facial expressions
Food images with different fat (***n*** = 100) [51]; fish burger packages (***n*** = 40) [54]; breakfast meals videos (***n*** = 40) [67]	Facial expressions did not vary among food images and showed low intensity
Consumer familiarity (***n*** = 60) [66]; consumer familiarity (***n*** = 40) [94]	Some facial expressions weakly correlated with familiarity

**Table 5:** Effect of food products and consumer traits on the Electroencephalography (EEG) response of consumers.

Factors	Observed Effects and Relations with Electroencephalography
Green tea odours (***n*** = 10) [103]; flavoured chewing gums (***n*** = 9) [99]; different drinks (***n*** = 6) [104]; tastant solutions (***n*** = 10) [105]; umami tastants (***n*** = 20) [106]; sweet tastants (***n*** = 30) [107]; comfort and daily foods (***n*** = 13) [108]	Alpha significantly varied among different flavours and odours
Chewing gums (***n*** = 9) [99]; chewing gums (***n*** = 11) [100]	Alpha was significantly higher during mastication
Green tea odours (***n*** = 10) [103]; odours (***n*** = 9) [99]; different drinks (***n*** = 6) [104]	Beta significantly varied among different flavours and odours
Chewing gums (***n*** = 48) [101]; chewing gums (***n*** = 9) [99]	Beta significantly varied after and during mastication
Food odours (***n*** = 21) [109]; beers (***n*** = 30) [64]; cold and hot drinks (***n*** = 13) [102]	Theta significantly varied among products
Odours (***n*** = 9) [99]	Theta did not significantly vary among products
Food images varying caloric content (***n*** = 19) [110]	Beta significantly varied among food images
Consumer familiarity (***n*** = 32) [108], food neophobia (***n*** = 43) [111]	Alpha was significantly affected by familiarity and food neophobia
Obesity and non-obesity (***n*** = 12) [112]	Beta was significantly affected by the weight status

**Table 6:** Effect of food products and consumer traits on the functional Magnetic Resonance Imaging (fMRI) response of consumers.

Factors	Observed Effects and Relations with Functional Magnetic Resonance Imaging
Milkshakes with different fat content (***n*** = 14) [116]; milkshakes with different fat content (***n*** = 14) [117]; milkshakes with different fat and sugar content (***n*** = 22) [118]; taste solutions (***n*** = 19) [119]; food and non-food images (***n*** = 16) [120]; food odours (***n*** = 23) [121]; odours [122]	Activity in Orbitofrontal Cortex (OFC) varied among products
Milkshakes with different fat content (***n*** = 14) [116]	Activity in lateral hypothalamus varied among products
Milkshakes with different fat content (***n*** = 14) [116]; taste solutions (***n*** = 19) [119]; food and non-food images (***n*** = 16) [120]; food odours [122]	Activity in amygdala varied among products
Milkshakes with different fat content (***n*** = 14) [116]	Activity in Somatosensory Cortex varied among products
Food rejection (***n*** = 15) [123]; consumer familiarity (***n*** = 10) [124]	Different brain activation depending on the consumer traits (food rejection or familiarity)

## Data Availability

No new data were created or analysed in this study. Data sharing is not applicable to this article.

## Abbreviations

The following abbreviations are used in this manuscript:

GSR	Galvanic Skin Response
HR	Heart Rate
PS	Pupil Size
ANS	Autonomic Nervous System
EEG	Electroencephalography
fMRI	Functional Magnetic Resonance Imaging
OFC	Orbitofrontal Cortex
VTA	Ventral Tegmental Area
